# Cold and Hot Extremozymes: Industrial Relevance and Current Trends

**DOI:** 10.3389/fbioe.2015.00148

**Published:** 2015-10-20

**Authors:** Felipe Sarmiento, Rocío Peralta, Jenny M. Blamey

**Affiliations:** ^1^Swissaustral USA, Athens, GA, USA; ^2^Fundación Científica y Cultural Biociencia, Santiago, Chile

**Keywords:** extremophiles, extremozymes, cold-adapted enzymes, psychrophiles, thermophiles, hyperthermophiles

## Abstract

The development of enzymes for industrial applications relies heavily on the use of microorganisms. The intrinsic properties of microbial enzymes, e.g., consistency, reproducibility, and high yields along with many others, have pushed their introduction into a wide range of products and industrial processes. Extremophilic microorganisms represent an underutilized and innovative source of novel enzymes. These microorganisms have developed unique mechanisms and molecular means to cope with extreme temperatures, acidic and basic pH, high salinity, high radiation, low water activity, and high metal concentrations among other environmental conditions. Extremophile-derived enzymes, or extremozymes, are able to catalyze chemical reactions under harsh conditions, like those found in industrial processes, which were previously not thought to be conducive for enzymatic activity. Due to their optimal activity and stability under extreme conditions, extremozymes offer new catalytic alternatives for current industrial applications. These extremozymes also represent the cornerstone for the development of environmentally friendly, efficient, and sustainable industrial technologies. Many advances in industrial biocatalysis have been achieved in recent years; however, the potential of biocatalysis through the use of extremozymes is far from being fully realized. In this article, the adaptations and significance of psychrophilic, thermophilic, and hyperthermophilic enzymes, and their applications in selected industrial markets will be reviewed. Also, the current challenges in the development and mass production of extremozymes as well as future prospects and trends for their biotechnological application will be discussed.

## Historical Background and Commercial Prospects of Enzymes

Enzymes are nature’s own catalysts. They are proteins which accelerate the rate and specificity of chemical reactions by reducing the required activation energy. Unknowingly, enzymes have been used as biotechnological tools since ancient times for the production of food and alcoholic beverages, but only recently have significant knowledge and understanding of enzymes been cultivated. Early ideas about enzymes and bio-catalytic processes started to take form during the seventeenth and eighteenth centuries; however, the first major breakthroughs were not achieved until the nineteenth century. In 1833, the first enzyme was discovered, diastase (Payen and Persoz, 1833), now known as amylase. The term enzyme was not adopted until 44 years later in 1877 by the German scientist Kühne (1877).

Nearly 40 years later, the first enzymatic preparation for a commercial application was developed by Otto Rohm in 1914. He isolated trypsin from animal pancreases and added it to washing detergents to degrade proteins. It was not until the 1960s that enzymatic biocatalysis became a viable industrial option with the mass production of microbial proteases for use in washing powders. Industrial enzymes have since evolved into a multibillion dollar global market. The global market for industrial enzymes is expected to reach $7 billion by 2018 with a compound annual growth rate (CAGR) of 8.2% from 2013 to 2018 (Dewan, 2014). In addition, the global specialty enzymes market is forecasted to reach about $4 billion by 2018^1^. This market will likely continue to grow for the foreseeable future as a result of advancements in the biotechnology industry, the continued need for cost-efficient manufacturing process, and calls for greener technologies.

The enzyme market is becoming increasingly dynamic. Recent enzyme discoveries and developments in genetics and protein engineering have increased the reach of enzymes in industry. Indeed, industrial catalysis is increasingly dependent on enzymes. Enzymes have become important tools for diverse industrial markets, such as food and beverage, animal feed, detergents, and technical enzymes, including biofuels, leather, pulp and paper, and textile markets. Specialty enzyme use is also growing in markets, such as diagnostics, pharmaceuticals, and research and development.

## From Enzymes to Extremozymes

Due to the intrinsic characteristics of enzymes, they have influenced almost every industrial market and their demand has constantly increased over the years. These natural catalysts are fast, efficient, and selective, in addition to producing low amounts of by-products. They are also fully biodegradable molecules, resulting in a low environmental impact (Rozzell, 1999) and a greener solution to many industrial challenges.

Since the first steps toward commercial scale enzyme production in the 1960s, when Novozymes (Bagsvaerd, Denmark) began marketing and mass producing proteases from *Bacillus* for use in detergents, microorganisms have played a pivotal role in the discovery and development of novel enzymes for industrial applications. The diversity, and unique properties of microbial enzymes, e.g., consistency, reproducibility, high yields, and economic feasibility among others, have elevated their biotechnological interest and application to different industrial areas (Gurung et al., 2013). However, the vast majority of current industrial processes are performed under harsh conditions, including extremely high and low temperatures, acidic or basic pHs, and elevated salinity. Standard enzymes have specific requirements for maximal function, performing optimally in narrow ranges of physical and chemical conditions. These requirements are quite different from industrial processing settings, where standard enzymes are easily denatured. In many cases, traditional chemical solutions are still the only viable option under such harsh conditions. There is a clear need for more sustainable and environmentally friendly methods to replace the current potentially harmful chemical processes. The identification of more novel enzymes with properties that can cope with industrial processing conditions is the key to the future of biocatalysis.

Extremophiles are organisms, mainly microorganisms, which belong to the domains *Archaea* and *Bacteria*. They thrive in environmental conditions considered by human standards to be extreme. Extremophiles grow and reproduce under high temperatures in hot springs or thermal vents, low temperatures in glaciers or the deep sea, acidic and basic pHs in industrial or mine wastewater effluents, high concentration of salts in salt lakes, and high levels of radiation and extreme desiccation in deserts among other physical or chemical extreme conditions in various ecological niches. For additional information about extremophiles and their specific characteristics and habitats, please visit the following reviews (Madigan and Marrs, 1997; Rothschild and Mancinelli, 2001; Canganella and Wiegel, 2011).

The following question then arises: how can these organisms survive, let alone thrive under such harsh conditions? The answer sounds simple at first: extremophiles evolved and adapted to their environments by developing unique mechanisms to keep their cellular components stable and active. However, these mechanisms are quite complex. For example, to cope with high concentrations of salt, extremophiles produce increased amounts of compatible solutes inside the cells or use ion pumps to keep an osmotic equilibrium; in the presence of low pH, they use proton pumps to keep an adequate pH inside the cell; and in the presence of low or high temperatures, they can modify the composition of their cytoplasmic membrane.

In many cases, enzymes from extremophiles have adapted to withstand extreme conditions on their own. These adaptations correspond to key changes in the amino acid sequence, which are translated into variations in the structure, flexibility, charge, and/or hydrophobicity of the enzymes. These changes do not follow a pattern or a specific trend. Extremophilic proteins display substantial variability in adaptations for similar extreme physical or chemical conditions (Reed et al., 2013).

These diverse adaptations allow extremozymes to expand the ranges of optimal enzyme performance enabling enzymatic biocatalysis under the enzymatically unfavorable conditions found in industrial processes. Through actively bioprospecting extreme environments and/or using genetic engineering, it is possible now to discover and develop extremozymes that can accommodate existing industrial processes or products. Extreme biocatalysis offers exciting opportunities to improve current enzyme technologies and represents a highly attractive, sustainable, cost-effective, and environmentally friendly option compared to chemical catalysis.

The present review focuses on the diverse adaptations and characteristics of psychrophilic, thermophilic, and hyperthermophilic enzymes and some of their current applications in select industrial and research areas.

## Cold Extremozymes: Industrial Relevance and Current Trends

### Psychrophilic microorganisms

More than three-quarters of our planet is comprised of environments which experience extremely low temperatures (≤15°C). It is not surprising, therefore, that the majority of Earth’s biomass is generated at temperatures below 5°C (Siddiqui and Cavicchioli, 2006). These habitats sustain a wide diversity of microorganisms, which naturally thrive in cold environments, known as psychrophilic microorganisms. They are adapted to grow, reproduce, and maintain their vital metabolic rates under such extreme conditions.

Psychrophiles are present in all three domains of life (*Archaea*, *Bacteria*, and *Eukarya*) and use a wide variety of metabolic pathways, including photosynthetic, chemoautotrophic, and heterotrophic pathways. By definition, psychrophiles are able to sustain growth in cold temperatures ranging from −20°C to +10°C. They are able to maintain an active metabolism at low temperatures, and many grow well at temperatures around the freezing point of water. The bacteria *Planococcus halocryophilus* Or1, isolated from high Arctic permafrost, grows and reproduces at −15°C, the lowest temperature demonstrated to date (Mykytczuk et al., 2013). Metabolically active bacteria have been reported at temperatures as low as −32°C (Bakermans and Skidmore, 2011), pushing the limits of life even further.

Psychrophiles face diverse challenges inherent to living in cold environments: low enzyme activity and low enzymatic rates, altered transport systems, decreased membrane fluidity, and protein cold-denaturation among others (D’Amico et al., 2006). To overcome these challenges, they have developed remarkable adaptations. For example, psychrophiles produce higher amounts of unsaturated and methyl-branched fatty acids and shorter acyl-chain fatty acids to increase membrane fluidity (Russell, 1997; Chintalapati et al., 2004). They also produce cold-shock proteins to aid in different cellular processes, such as protein folding or membrane fluidity (Phadtare, 2004), and antifreeze proteins that inhibit ice crystal growth. In addition, all components of their cells must be suitably adapted to cold temperatures, including cold-adapted enzymes that are extremely flexible and maintain high specific activities at low temperatures.

### Structural features and action mechanism of cold-adapted extremozymes

At low temperatures, the mean kinetic energy available for reactions is low and insufficient to overcome the energy barrier of activation for catalysis. This translates into reduced enzymatic activity. Proteins also tend to denature as temperatures drop because of a decrease in the availability of water molecules as the molecules become more ordered and less associated with proteins (Karan et al., 2012). Cold-adapted (or psychrophilic) enzymes have a combination of specific adaptations in their structural features that give them more flexible structures than mesophilic and thermophilic enzymes. This trait allows for high catalytic activity at low temperatures (Siddiqui and Cavicchioli, 2006), and thermolability. These adaptations correspond to specific genetic changes, which are a consequence of long-term selection. In recent reviews on psychrophilic enzymes, the following features were mentioned as important adaptations for keeping high flexibility and high activity at low temperatures: (a) decreased core hydrophobicity, but increased surface hydrophobicity, (b) changes in amino acid compositions, such as lower arginine/lysine ratio, more glycine residues for better conformational mobility, fewer proline residues in loops, but more in α-helices, and more non-polar residues on the protein surface, (c) weaker protein interactions, such as inter-domain and inter-subunit interactions, less disulfide bridges, fewer hydrogen bonds and other electrostatic interactions, and less/weaker metal-binding sites, (d) decreased secondary structures and oligomerization, but an increase in the number and size of loops, and (e) an increase in conformational entropy of the unfolded protein state (Feller, 2010; Cavicchioli et al., 2011). As a consequence of these special features, the reaction rate of psychrophilic enzymes tends to decrease more slowly compared to similar enzymes from mesophilic or thermophilic microorganisms when the temperature decreases (Feller, 2013). Collins and collaborators compared cold-adapted and mesophilic xylanases and demonstrated that cold-adapted enzymes are more active at low temperatures, but more thermolabile when the temperature increases (Collins et al., 2002).

### Biotechnological applications of cold extremozymes

In recent years, scientific and industrial efforts to discover and develop novel cold-adapted enzymes have increased substantially. The intrinsic characteristics of cold-adapted enzymes, high activity at low temperatures and thermolability, are extremely valuable for different biotechnological applications in a wide variety of industries from molecular biology, to detergency to food and beverage preparation (Marx et al., 2007; Cavicchioli et al., 2011; Feller, 2013). As a consequence, psychrophilic enzymes are replacing mesophilic enzymes in many different industrial processes.

There are several benefits to the application of cold-adapted enzymes in industry. Due to their high activity, the desired result from a biochemical reaction can be reached using smaller amounts of a psychrophilic enzyme compared to its mesophilic or thermophilic homologs under optimal conditions. This faster activity translates into savings in time, energy, and money for a particular reaction. In addition, due to their thermolability, cold-adapted enzymes can be selectively inactivated in a complex mixture by increasing the temperature of the reaction. This represents a distinct advantage when an enzyme is needed only for a certain period of time in a particular reaction, e.g., meat tenderizing by proteases, or to avoid an undesirable byproduct of that particular enzymatic reaction that is produced at higher temperatures. This characteristic allows for improved control and a higher effectiveness of the overall reaction and represents a clear advantage for the use of enzymes in various processes, including those used in molecular biology. Since psychrophilic enzymes function optimally at moderate and low temperatures, they can catalyze reactions at ambient temperatures eliminating the need for heat input into a system. This translates into the development of more cost-efficient processes. This particular trait is of the utmost importance to the detergent industry. It allows for the development of detergents effective at ambient temperatures that are necessary not only to reduce the environmental and economic impact of reducing washing temperatures but also to supply working products to people with limited access to warm water. Lastly, psychrophiles have adapted to extremely cold environments, with varying substrate availability, ecology, and other characteristics not found in mesophilic and thermophilic environments. Psychrophiles represent an interesting source of novel enzymes that may use different substrates or co-factors, and/or perform innovative reactions applicable to industry. Described below are the applications in selected industrial markets and some examples of in-development and commercially available cold-adapted enzymes (Table 1).

**Table 1:** **Examples of commercially available cold-active enzymes**.

Market	Enzyme	Commercially available	Uses
Molecular Biology	Alkaline phosphatases	Antarctic phosphatase (New England Biolabs Inc.)	Dephosphorylation of 5′ end of a linearized fragment of DNA
	Uracil-DNA N-glycosylases (UNGs)	Uracil-DNA *N*-glycosylase (UNG) (ArcticZymes), Antarctic Thermolabile UDG (New England Biolabs Inc.)	Release of free uracil from uracil-containing DNA
	Nucleases	Cryonase (Takara-Clontech)	Digestion of all types of DNA and RNA
Detergent	Lipases	Lipoclean^®^, Lipex^®^, Lipolase^®^ Ultra, Kannase, Liquanase^®^, Polarzyme^®^, (Novozymes)	Breaking down of lipid stains
	Proteases	Purafect^®^ Prima, Properase^®^, Excellase (Genencor)	Breaking down of protein stains
	Amylases	Stainzyme^®^ Plus (Novozymes), Preferenz^™^ S100 (DuPont), Purafect^®^ OxAm (Genencor)	Breakdown starch-based stains
	Cellulases	Rocksoft^™^ Antarctic, Antarctic LTC (Dyadic), UTA-88 and UTA-90 (Hunan Youtell Biochemical), Retrocell Recop and Retrocell ZircoN (EpyGen Biotech), Celluzyme^®^, Celluclean^®^ (Novozymes)	Wash of cotton fabrics
	Mannanases	Mannaway^®^ (Novozymes), Effectenz^™^ (DuPont)	Degradation of mannan or gum
	Pectate lyases	XPect^®^ (Novozymes)	Pectin-stain removal activity
Textile	Amylases	Optisize^®^ COOL and Optisize NEXT (Genencor/DuPont)	Desizing of woven fabrics
	Cellulases	Primafast^®^ GOLD HSL IndiAge^®^ NeutraFlex, PrimaGreen^®^ EcoLight 1 and PrimaGreen^®^ EcoFade LT100 (Genencor/DuPont)	Bio-finishing combined with dyeing of cellulosic fabrics
Food and beverages	Pectinases	Novoshape^®^ (Novozymes), Pectinase 62L (Biocatalysts), Lallzyme^®^ (Lallemand)	Fermentation of beer and wine, breadmaking, and fruit juice processing
Other	Catalase	Catalase (CAT), (Swissaustral)	Textile, research, and cosmetic applications

#### Cold Extremozymes Applied in Molecular Biology

The heat lability associated with cold-adapted enzymes is an essential feature for sequential enzymatic applications in a variety of processes, including many techniques used in molecular biology. One of the most commonly used enzymes in molecular biology is alkaline phosphatase (AP). This enzyme is used to dephosphorylate the 5′ end of a linearized fragment of DNA to prevent re-circularization when performing cloning techniques and also in many other applications. For many years, the only APs in the market were from *Escherichia coli* or calf intestine. Since these enzymes are heat-stable, it is necessary to carefully inactivate them using detergents; these detergents, however, can interfere with subsequent steps of this particular technique. In contrast, psychrophilic heat-labile AP can be easily inactivated in the same tube by a moderate heat treatment (~65°C). The first heat-labile AP was purified in 1984 from a bacterium isolated from Antarctica (Kobori et al., 1984). A recombinant AP isolated originally from another Antarctic bacterium (Rina et al., 2000), and further improved through directed evolution (Koutsioulis et al., 2008) is commercially available as Antarctic phosphatase by New England Biolabs (Ipswich, MA, USA). The first commercially available cold-adapted AP was developed from the arctic shrimp *Pandalus borealis* by ArcticZymes (Tromsø, Norway) in 1993. ArcticZymes launched a recombinant version of this AP in 2010. A novel psychrophilic AP was recently isolated from a metagenomic library constructed using ocean-tidal flat sediments from the west coast of Korea (Lee et al., 2015). This enzyme exhibits similar characteristics and comparable efficiency to other commercially available APs.

There are other examples of cold-adapted enzymes used in molecular biology: (a) Uracil-DNA N-glycosylase (UNG) catalyzes the release of free uracil from uracil-containing DNA. UNG is used to control carry-over contamination in PCR and RT-PCR, in site-directed mutagenesis and in SNP genotyping among other applications. ArcticZymes produces a commercially available cold-adapted UNG enzyme that is completely and irreversibly inactivated by heat treatment (55°C). This enzyme is derived from Atlantic cod (*Gadus morhua*) and its recombinant version produced in *E. coli* is available commercially (Lanes et al., 2002). A novel heat-labile UNG was discovered by mining the genome of the bacterium *Psychrobacter* sp. HJ147. This enzyme was cloned and expressed in *E. coli*. It functions with an optimal temperature of 20–25°C and a half-life of 2 min at 40°C (Lee et al., 2009). Additionally, New England Biolabs offers Antarctic Thermolabile UDG, a recombinant UNG enzyme produced in *E. coli* from a psychrophilic marine bacterium with an inactivation temperature above 50°C. (b) Double strand-specific DNase allows for the digestion of double-stranded DNA without any effect on single-stranded DNA molecules, such as primers or probes. This enzyme is used to decontaminate PCR mixes or to remove genomic DNA from RNA preparations. A heat-labile version of this enzyme (inactivated at 55°C) isolated from shrimp (*P. borealis*) and further genetically engineered is offered from ArcticZymes. A recombinant version produced in *Pichia pastoris* is offered by Affymetrix USB (Santa Clara, CA, USA) and is inactivated after exposure to 70°C for 25–30 min. (c) Cryonase, a recombinant cold-active nuclease offered by Takara-Clontech (Mountain View, CA, USA), is derived from a psychrophilic strain of *Shewanella* sp. It is produced in *E. coli* and can digest all types of DNA and RNA (single-stranded, double-stranded, linear, or circularized). This enzyme is active even when samples are left on ice and can only be completely inactivated after a 30 min incubation at 70°C (Awazu et al., 2011).

The introduction of new cold-adapted enzymes to the molecular biology market would greatly benefit this field and would be an ideal application for psychrophiles. For example, cold-active recombinases are part of the new recombinase polymerase amplification (RPA) PCR kits being developed by TwistDx (Cambridge, UK). RPA is an isothermal amplification method that utilizes a recombinase to facilitate the insertion of oligonucleotide primers onto their complement in a double-stranded DNA molecule. The use of opposing primers allows for the exponential amplification of a defined region of DNA in a manner similar to PCR. This technology has been successfully applied in the detection of HIV and protozoan parasite like *Plasmodium falciparum*, the causal agent of malaria.

DNA ligases are commonly utilized tools in molecular biology for catalyzing the formation of phosphodiester bonds in nicked double-stranded DNA molecules. Currently, the market of DNA ligases is dominated by recombinant versions of bacteriophage-derived DNA ligases, such as the T4 and T7 ligases, and *E. coli* DNA ligases. All of these function at temperatures above 15°C. At these elevated temperatures, residual nucleases may interfere with the ligation process. The introduction of cold-adapted DNA ligases from psychrophiles may offer a novel advantage by maintaining a high specific activity at temperatures low enough that nucleases are no longer active. A novel DNA ligase from the psychrophile *Pseudoalteromonas haloplanktis* has been cloned, overexpress and characterized. This DNA ligase displays activity at temperatures as low as 4°C (Georlette et al., 2000). Proteinase k is another cold-adapted enzyme with potential applicability to molecular biology. This enzyme, which belongs to the serine proteases family, is normally used to degrade unwanted enzymes from crude extracts or enzymes used in previous steps of an experimental protocol. Current commercially available proteinase k needs to be inactivated by chemical removal (phenol-chloroform extraction/ethanol precipitation) which can lead to sample loss and contamination, or heat inactivation at high temperatures (95°C) which can denature the target product. A cold-adapted thermolabile proteinase k-like serine protease would be highly active at low temperatures and could be inactivated with mild heat (45–60°C), increasing the overall efficiency of the experiments and reducing contamination risk. There is currently no cold-adapted DNA ligase or proteinase k is commercially available.

#### Cold Extremozymes Applied in Detergent Market

For many years, enzymes have been common components of detergent formulations in developed countries. The global market for detergent enzymes in 2013 was valued at approximately $1 billion and is expected to reach $1.8 billion by 2018 with a CAGR from 2013 to 2018 of 11.3% (Dewan, 2014). Detergent enzymes will represent 25–30% of the global market of enzymes for industrial applications. The current trend in the detergent market is cold-water detergents that can work as efficiently as common detergents but at lower temperatures. The application of cold-water detergents would allow for reduced energy consumption and carbon dioxide emissions as well as improved fabric protection. Despite the appeal of this idea, it has taken time to become a widely spread option. Hot water remains the preferred standard method for cleaning clothes. However, recent efforts to discover and develop novel enzymes that can work efficiently at cold temperatures are helping to change the cleaning industries awareness and creating an excellent opportunity for the application of cold-wash detergents. The percentage of global cold-water washing machine loads increased from 38 to 53% from 2010 and 2014. Additionally, one of the main goals of Proctor & Gamble and Unilever, the largest producers of detergent technologies, for 2020 is to have 70% of all machine washer loads done in cold water, reducing the energy impact and greenhouse gas emissions of laundry activities^2^^,^^3^.

Several different cold-adapted enzymes are of interest for improving the efficacy of cold-water household and industrial laundry and dishwasher detergents, some of the most important are described below.

Lipases catalyze the hydrolysis of fats (lipids) and remove fatty stains (butter, oil, and sauces) from fabrics. Novozymes, one of the worldwide largest producers of enzymes, has developed Lipoclean^®^, a cold-adapted lipase that targets stains from triglycerides. Lipoclean^®^ is active at low temperatures (~20°C) and is very stable in multienzyme solutions. In addition, Novozymes has developed two other alternative lipases: Lipex^®^ and Lipolase^®^ Ultra, which perform optimally in low–moderate temperatures. Research for the discovery and development of novel cold-adapted lipases from psychrophilic microorganisms is booming because of the useful application of lipases in detergents and the trend toward cold-washing detergency (Park et al., 2009; Xuezheng et al., 2010; Cheng et al., 2011; Litantra et al., 2013; Jiewei et al., 2014; Ji et al., 2015). A high-performance lipase from *Pseudomonas stutzeri* PS59 was recently identified for use in detergent formulations. This lipase has optimal activity at 20°C, pH 8.5 and in the presence of different surfactants and oxidizing agents (Li et al., 2014).

Proteases are enzymes that catalyze the hydrolysis of peptide bonds that link amino acids together, digesting proteins into shorter fragments. They fulfill an important role in the detergency industry by breaking down protein stains, such as blood, egg, grass, cocoa, and human sweat (Joshi and Satyanarayana, 2013). Bacterial proteases were first used in laundry detergents in 1959 for the development of the detergent Bio 40 from Gebruder Schnyder-Biel. Novozymes has developed several cold-adapted proteases for different detergency applications. Kannase^®^ (or Liquanase^®^), released in 1998, was one of the first proteases for washing laundry at cold temperatures, between 10 and 20°C. It corresponds to a non-specific protease that belongs to the serine proteases group. Novozymes also released, Polarzyme^®^, another variation of a serine protease for use when hand washing laundry using cold water. Polarzyme^®^ maintains high activity in a broad range of temperatures from 5 to 60°C. The enzyme production company Genencor (Palo Alto, CA, USA), a division of DuPont (Wilmington, DE, USA), developed two cold-adapted proteases for laundry detergents, Purafect^®^ Prime and Properase^®^, with optimal activity for soil stains removal at temperatures between 20 and 40°C. Genencor also developed Excellase for dishwashing at low or moderate temperatures. Recent research has focused in discovering novel cold-adapted proteases in psychrophilic microorganisms derived from Arctic and Antarctic ecosystems. A recent study screened and identified 68 protease-producing bacterial strains from the Arctic (Chen et al., 2013a). A novel serine protease was isolated from *Glaciozyma antarctica* strain PI12, cloned and expressed in *P. pastoris*, and displays high activity at 20°C (Alias et al., 2014). These new proteases represent an interesting alternative to currently utilized cold-adapted proteases. In recent years, two patents for cold-adapted bacterial proteases have been filed (Zhang et al., 2013; Asenjo et al., 2014b).

Amylases are widely used enzymes in detergent formulations. They breakdown starch-based stains from different foods, such as cereals, fruits, BBQ sauce, gravy, pasta, and potatoes. The most commonly used amylase class in detergency is α-amylase, which hydrolyzes the 1,4-α-glucosidic bond found in starch and decomposes it into water-soluble dextrins and oligosaccharides (Hmidet et al., 2009). Currently, there are few cold-active amylases on the market, but their applications have increased over time. In 2004, Novozymes released Stainzyme^®^ an amylase that is effective at temperatures between 30 and 70°C. Even though this enzyme is not classified as a psychrophilic enzyme because of the temperature range for its activity, it was the first recognized amylase for moderate/low-temperature washing. In 2007, Novozymes released an improved version of this enzyme, Stainzyme^®^ Plus, which displays an in-wash bleach tolerance and works efficiently at even lower temperatures (~20°C). DuPont Industrial Biosciences developed Preferenz™ S100, an amylase enzyme that enables high-performance laundry at 16°C. Consistent effort is being made to develop a new generation of cold-active amylases from psychrophilic/psychrotolerant microorganisms. For instance, in 2013 a novel cold-active, detergent-stable α-amylase from *Bacillus cereus* GA6 was characterized (Roohi et al., 2013). This extracellular enzyme is stable and active at low temperatures (4–37°C, with optimal activity at 22°C) and in alkaline pH (pH 7–11) and demonstrates good compatibility with commercial laundry detergents. Another amylase with potential applicability in detergency was isolated from the marine bacterium *Zunongwangia profunda* in 2014 (Qin et al., 2014). The recombinant *E. coli* form is active in a wide range of temperatures, from 0 to 35°C, maintaining 39 and 46% of activity at 0 and 5°C, respectively.

Cellulases are hydrolytic enzymes that breakdown cellulose into monosaccharides or “simple sugars” by catalyzing the degradation of β-1,4-glycosidic bonds. They are used in detergents for color and brightness care as they reduce the build-up of fuzz and pills in knitted garments. Normal use and repeated washing of cotton fabrics breaks down the cotton fibers (made of cellulose), causing them to trap dirt and soil. Cellulases digest the broken cellulose fibers removing the captured dirt particles. Additionally, cellulase removes β-glucan stains from oat products, such as cookies, cereals and snack bars. Currently, there are several available cellulases that are marketed for cold-washing, but these cellulases are not typically classified as a psychrophilic or even psychrotolerant enzyme. They display optimal activity between 30 and 60°C, but are active at lower temperatures as well. Examples of these enzymes includes: Rocksoft™ Antarctic and Antarctic LTC from Dyadic International (Jupiter, FL, USA), UTA-88 and UTA-90 from Hunan Youtell Biochemical (Shangai, China), and Retrocell Recop and Retrocell ZircoN from EpyGen Biotech (Dubai, UAE) (Kasana and Gulati, 2011). Truly cold-adapted cellulases were developed by Novozymes: Celluzyme^®^, which is derived from the fungus *Humicola insolens* and is active at low temperatures (~15°C), and Celluclean^®^ a mix of cellulases for low-temperature washing.

In addition to these common detergent enzymes, the laundry and dishwashing industry is actively looking for other cold-active enzymes, such as mannanases and pectinases. Mannanase are used to degrade mannan or gum, a carbohydrate used extensively in food and personal care products. This enzyme hydrolyzes the β-1,4 bonds in mannose polymers, breaking them down into smaller more soluble carbohydrates. Few cold-active mannanases are currently commercially available: Mannaway^®^ (Novozymes) can be used at temperatures as low as 20°C and Effectenz™ (DuPont) has optimal activity at 30°C. Pectinases are a group of enzymes that hydrolyzes pectin, a polysaccharide found in a variety of fruits, fruit juices, marmalades, and tomato sauces. This group of enzymes provides pectin-stain removal activity for detergent formulations. Cold-active pectinases are a current target for the detergent industry. XPect^®^ (Novozymes) was released into the European market in 2010. XPect^®^ is a cold-active pectate lyase that cleaves the α-1,4 glycosidic bonds of polygalacturonic acid into water-soluble “simpler sugars” optimally at 20°C.

Despite the several advances made in the development of cold-active enzymes for the detergent industry in recent years, there is a clear need for still more psychrophilic/psychrotolerant enzymes that can perform optimally under the current cold-washing practices. Enzymes that can work optimally at low temperatures (15–25°C), but maintain a high activity through a wide range of temperatures (between 5 and 60°C), and stay active in the presence of surfactants and alkaline pH are the future for this industry.

#### Cold Extremozymes Applied in the Food and Beverages Market

For hundreds of years, enzymes have been used in foods and beverages. In the dairy industry, enzymes are used for cheese production and in the preparation of dairy products; in the baking industry, enzymes improve the quality of bread; and in the beverage industry, enzymes are used to maintain wine color and clarity, and to reduce the sulfur content. Some industrial enzymes can also be used to enhance filterability and improve the flavor of final products. Food and beverage enzymes make up one of the biggest markets for industrial enzyme applications. In 2011, this market was worth $1.2 billion and is expected to grow at a CAGR of 5.1% from 2013 to 2018, reaching a worldwide value of $1.7 billion by 2018 (Dewan, 2014). Europe is predicted to be the most active region.

Food enzymes can be divided into categories depending on their preferred use: food additives, food ingredients, and processing aids. In general, enzymes functioning as food additives are used to preserve flavor or enhance taste and appearance. For example, invertase and lysozyme are used as stabilizers and preservatives, respectively. Both enzymes are authorized by the European Commission (EC) as food additives. Enzymes used as food ingredients are added to increase nutritional value. Enzymes functioning as processing aids are added to food preparations for technical reasons; however, they do not have a function in the final food product. Lactase from *Kluyveromyces lactis*, for instance, is used to hydrolyze the lactose in milk for the manufacture of lactose-free products (Mateo et al., 2004). Other enzymes, such as α-amylases, peptide hydrolases, lipases, and catalases are some examples of enzymes that are added during food processing to confer specific characteristics to the food. These enzymes are inactivated in the final product.

The current food and beverage industry trend is to replace high-temperature processes with low-temperature processes. Low-temperature processing provides economic and environmental advantages. Some of the benefits of low-temperatures processing are energy savings, prevention of contamination and spoilage, retention of labile and volatile flavor compounds, minimization of undesirable chemical reactions that may occur at higher temperatures, and a higher degree of control over cold-active enzymes since they can be inactivated at high temperatures (Horikoshi, 1999; Pulicherla et al., 2011). As a consequence, a myriad of cold-adapted enzymes have been discovered and developed for use in the food and beverages market. A few examples are discussed below.

As in the detergent industry, amylases have applications in several food and beverage industry processes including, fermentation of beer and wine, bread making and fruit juice processing. All these applications are based on the degradation of starch into less complex sugars. The vast majority of amylases currently in use in the food and beverages industry are thermophilic, but this is changing because of the benefits of using cold-active enzymes. The first cold-adapted α-amylase studied was isolated from an Antarctic bacterium, *Alteromonas haloplanktis*, and has been successfully expressed in the mesophilic host *E. coli* (Feller et al., 1998). This enzyme and other cold-active α-amylases, such as the extracellular α-amylase from the bacterium *Microbacterium foliorum GA2* isolated from the Gangotri glacier (Kuddus, 2012) or the cold-active α-amylase from marine bacterium *Z. profunda* (Qin et al., 2014), represent good candidates for application in this particular industry. A patent relating to a variant of the parent α-amylase from *Bacillus licheniformis* displaying increased specific activity at temperatures from 10 to 60°C was granted to Novozymes in 2004 (Borchert et al., 2004).

Among enzymes in the food industry, β-galactosidases or lactases are highly valuable to the food industry. Lactases, as mention above, hydrolyze lactose into galactose and glucose, allowing for the production of lactose-free foodstuffs. The development of cold-active β-galactosidases is an intriguing idea for many companies because of the potential applications cold-active β-galactosidases would allow lactose degradation milk and other dairy products during refrigeration, cheese whey bio-remediation, and sweetener production. Several companies, such as Biocatalysts (Wales, UK), DSM/Valley Research (South Bend, IN, USA), DuPont/Danisco (Wilmington, DE, USA), and Enzyme Development Corporation (New York, NY, USA), offer β-galactosidases or lactases which are not psychrophilic enzymes but show certain activities at low temperatures. A recent report compared several commercially available food-grade β-galactosidases, and demonstrated that those enzymes are sufficiently active in milk at refrigeration temperatures to enable the lactose hydrolysis process (Horner et al., 2011). However, novel cold-active β-galactosidases could simplify and reduce the cost of manufacturing lactose-free products. A cold-active β-galactosidase isolated from a marine psychrophilic bacterium was recently characterized. This β-galactosidases hydrolyzed around 80% of the lactose in raw milk at 20°C and pH 6.5 (Ghosh et al., 2012; Pulicherla et al., 2013). In 2012, a patent was granted to Stougaard and Schmidt (2010) for a cold-active β-galactosidase with stable enzymatic activity at temperatures <8°C. A company called Damhert Nutrition (Heusden-Zolder, Belgium) is currently using β-galactosidase as the first step for tagatose production, a novel sweetener that is obtained from lactose. In this process, lactose is broken down into galactose and glucose by β-galactosidase, subsequently, galactose is enzymatically transformed into tagatose. A recent publication demonstrated the benefits of using a cold-active β-galactosidase from the Antarctic marine bacterium *P. haloplanktis* for lactose hydrolysis at low temperatures (refrigeration conditions) from whey permeate and its potential applicability in tagatose production (Van de Voorde et al., 2014).

Pectinases are an integral part of the food industry. These enzymes catalyze the degradation of the plant carbohydrate pectin and are used in several food-related applications, like fruit juice processing for clarification and viscosity reductions and vinification and extraction of natural oils (Adapa et al., 2014). Today, most commercially available pectinases are derived from mesophilic organisms, but the current trend toward processing foods under low-temperature conditions is opening doors for the development of new cold-adapted pectinases. Several pectinases for use in the food and beverages industry are commercially available, but none are classified as psychrophilic enzymes. However, there are a couple of pectinase products that show activity at low temperatures. These include, Novoshape^®^ (Novozymes), a pectinmethylesterase produced by a genetically modified strain of the fungus *Aspergillus oryzae*, and Pectinase 62L (Biocatalysts Ltd.), which is composed of a mix of polygalacturonase and pectin lyase derived from *Aspergillus sp*. Novoshape^®^ and Pectinase 62L exhibit a temperature range for activity between 10 and 60°C. Lallemand (QC, Canada) produces Lallzyme^®^ a set of pectinase mixes (polygalacturonase, pectin esterase and pectin lyase) from the fungus *Aspergillus niger*. These mixes possess activity between 5 and 20°C and are used for the clarification of juices, musts, and wines. Additional information about the current state of cold-active pectinase research for the food industry and advances in this field can be found in a recent review by Adapa et al., 2014.

In bread making, xylanases convert the insoluble hemicellulose of dough into soluble sugars before baking, producing a fluffy but strong dough and voluminous loaves of bread with soft and elastic properties. Since these properties are obtained at low temperatures, before baking the bread, cold-active xylanases may provide a significant benefit. Despite their potential applicability, cold-active xylanases have been poorly studied and many of the xylanases used in industry today appear to be mesophilic or thermophilic (Collins et al., 2005). A recent report demonstrated that three psychrophilic xylanases from *P. haloplanktis* TAH3A, *Flavobacterium* sp. MSY-2 and one from an unknown bacterial origin effectively improved dough properties and final bread volume (up to 28%) when compared to mesophilic xylanases from *Bacillus subtilis* and *Aspergillus aculeatus* (Dornez et al., 2011). Several novel cold-adapted xylanases from different organisms have been reported in the last years (Wang et al., 2012; Chen et al., 2013b; Del-Cid et al., 2014), including one patented by Asenjo et al. (2014a).

#### Other Cold-Adapted Enzymes

Many other cold-active enzymes have further applications within the textile industry. Optisize^®^ COOL amylase (Genencor-DuPont) allows for the desizing of woven fabrics at low temperatures, Primafast^®^ GOLD HSL cellulase (Genencor-DuPont) can be applied during the bio-finishing of cellulosic fabrics, and IndiAge^®^ NeutraFlex cellulase allows low-temperature enzymatic stonewashing at a neutral pH.

Recently, the gene that codes for the psychrotolerant enzyme catalase (CAT) from a psychrotolerant microorganism belonging to the *Serratia* genus was successfully cloned and expressed in *E. coli*. CAT is a very active and extremely efficient antioxidant enzyme even at low temperatures. This enzyme is involved in trapping reactive oxygen species generated by oxidative stress. Psychrotolerant catalase was expressed as a recombinant enzyme and showed excellent catalytic properties and stability. This enzyme kept 50% of its activity after 7 h of 50°C exposure and is active in a wide range of temperatures from 20 to 70°C. Due to these unique characteristics, it can be applied in the textile, research, cement, and cosmetic industries. This enzyme is now commercially available through Swissaustral Company^4^ (Athens, GA, USA).

## Hot Extremozymes: Industrial Relevance and Current Trends

### Thermophilic and hyperthermophilic microorganisms

Thermophiles and hyperthermophiles are defined as organisms that not only survive but thrive at high temperatures. Thermophiles can be found in environments with temperatures over 50°C, and hyperthermophiles in environments over 80°C. To date, the highest temperatures known to sustain thermophilic microbial life are 113°C for the chemolithoautotrophic archaea *Pyrolobus fumarii* (Blochl et al., 1997; Cowen, 2004) and 122°C for the methanogenic hyperthermophile *Methanopyrus kandleri* (Takai et al., 2008). Thermophiles and hyperthermophiles are found in environments like hot springs and deep sea vents where temperatures easily surpass 100°C. Other habitats include geysers and highly geothermal volcanic areas.

Numerous thermophilic prokaryotes have been identified and thoroughly studied. The majority of thermophilic prokaryotes are from the domain Archaea, but some representatives from the domain Bacteria have also been identified (Huber and Stetter, 1998; Klenk et al., 2004). It has been suggested that hyperthermophiles are the most deeply branching organisms on the phylogenetic tree, making them among the most ancient known organisms (Di Giulio, 2005).

Sulfate-dependent organisms are some of the most common thermophiles and hyperthermophiles. Sulfate reduction, as found in the hyperthermophilic archaea *Archaeoglobus fulgidus* (Beeder et al., 1994; Klenk et al., 1997), is thought to be one of the most ancient energetic pathways (Wagner et al., 1998; Roychoudhury, 2004). Many of these organisms are from the archaeal phylum *Crenarchaeota* (Achenbach-Richter et al., 1987; Friedrich, 1998). Other anaerobic metabolic pathways used by (hyper)thermophiles include the production of methane from hydrogen and carbon dioxide, such as in the case of *Methanocaldococcus jannaschii* (Bult et al., 1996).

Sulfate oxidation also occurs in organisms that thrive in these extreme conditions. Examples of sulfate and, in some cases, elemental sulfur-oxidizing organisms are found within the *Sulfolobus* family (Brock et al., 1972; Kletzin et al., 2004). The oxidation of organic compounds and metals has also been reported and can be associated with low pH conditions as well (Larsson et al., 1990).

### Structural features and action mechanism of hot extremozymes

A great deal of attention has been focused on proteins from (hyper)thermophiles as thermostable enzymes have gained importance in biotechnological processes. Comparative studies on mesophilic and thermophilic enzymes and other proteins have shown that in most cases, amino acid sequences are highly conserved, although certain changes in composition have been observed. Studies of the glutamate dehydrogenase from *Pyrococcus furiosus*, which grows at 105°C, compared with mesophilic versions of the enzyme have shown a higher content of hydrophobic amino acids and a decrease in the content of polar and charged amino acids. A drop in the number of glycine residues was also observed (Maras et al., 1994). These results are not surprising when considering how thermodynamic factors influence protein stability.

Highly dense packing structures with tight hydrophobic cores minimize solvent interaction with this core and therefore, have a stabilizing effect. Since protein stability is dependent on small non-covalent forces, a high degree of cooperation among these hydrophobic interactions is expected. Another factor of increased thermostability is a decrease in the protein surface area in proportion to protein size. This has been observed in proteins from *P. furiosus*. Enzymes from *P. furiosus* have highly packed hydrophobic cores with many ionic pairs and buried atoms to help diminish unfavorable solvent interactions (Rice et al., 1996; Kumar and Nussinov, 2001).

When comparing 3D structures of (hyper)thermophilic proteins to their mesophilic counterparts, a highly conserved structure has been observed. This suggests the conserved residues have some functional significance. The majority of differences can be observed in the region of the protein that interacts with solvent: differences in the sizes of helices, beta sheets and ionic modifications in terminal portions (Ladenstein and Antranikian, 1998; Kumar and Nussinov, 2001). Again, tight packaging is observed, but this does not alter the final conformation.

### Biotechnological applications of hot extremozymes

From an industrial viewpoint, (hyper)thermophilic enzymes possess certain advantages over their mesophilic counterparts. These enzymes are active and efficient under high temperatures, extreme pH values, high substrate concentrations and high pressure. They are also highly resistant to denaturing agents and organic solvents. In addition, hot extremozymes perform faster reactions and are easier to separate from other heat-labile proteins during purification steps.

Due to their overall activity and stability at high temperatures, (hyper)thermophilic enzymes are attractive for several important industrial activities. It is difficult, however, to obtain pure enzymes from source microorganisms and yields are typically low when cultivated on a large scale for industrial applications. Efforts to overcome these problems have focused on cloning and expressing (hyper)thermophilic enzymes in mesophilic hosts. Ideally, this can be done without losing their activity and thermostability. Thermostable enzymes expressed in mesophilic hosts can typically be purified easily and the degree of purity obtained is suitable for industrial applications. The availability of an increasing number of hot extremozymes has opened new possibilities for industrial processes. There is a high availability of hot extremozymes with applications in several markets. This section of the review will focus on two established, but growing markets for their application, food and beverages and pulp and paper. Other markets, such as feed and biofuels, will not be covered here due to the large number of enzymes available and the complexity of these markets. Select examples of in-development and commercially available hot extremozymes are listed below (Table 2).

**Table 2:** **Examples of commercially available thermostable enzymes**.

Market	Enzyme	Commercially available	Uses
Food and beverages	Amylases	Avantec^®^, Termamyl^®^ SC, Liquozyme^®^, Novamyl^®^, Fungamyl^®^ (Novozymes), Fuelzyme^®^ (Verenium), AlphaStar PLUS (Dyadic)	Enzymatic starch hydrolysis to form syrups. Applied in processes, such as baking, brewing, preparation of digestive aids, production of cakes and fruit juices
	Glucoamylases	Spirizyme^®^ (Novozymes)	Used on liquefied starch-containing substrates
	Glucose (xylose) isomerases	Sweetzyme^®^ (Novozymes)	Isomerization equilibrium of glucose into fructose
	Proteases	Protease PLUS	Applied in brewing to hydrolyze most proteins
	Amyloglucosidases	GlucoStar PLUS (Dyadic)	Used in processing aids
	Xylanases, cellulases, pectinases, mannanases, β-xylosidases, α-l-arabinofuranosidases, amylases, protease, other	CeluStar XL, BrewZyme LP, Dyadic Beta Glucanase BP CONC, Dyadic xylanase PLUS, Xylanase 2XP CONC, AlphaStar CONC and Protease AP CONC. (Dyadic), Panzea BG, Panzea 10X BG, Panzea Dual (Novozymes), Cellulase 13P (Biocatalysts)	Hydrolyis of hemicellulose and cellulose to lower molecular weight polymers in brewing
	Lipases and xylanases	Lipopan^®^ and Pentopan^®^ (Novozymes)	Obtaining of stronger dough in bakery
	Glucose oxidases	Gluzym^®^ (Novozymes)	Used to obtain stronger gluten in bakery
Pulp and paper	Xylanases	Luminase^®^ PB-100 and PB-200 (Verenium), Xylacid^®^ (Varuna Biocell), Xyn 10A (Megazyme)	Bio-bleaching
	Laccases	Laccase (Novozyme)	Bio-bleaching
	Lipases and esterases	Lipase B Lipozyme^®^ CALB L, Lipase A NovoCor^®^AD L, Resinase^™^ HT and Resinase A2X (Novozymes), Optimyze^®^ (Buckman Laboratories)	Pitch control
	Cellulases/hemicellulases preparations	FibreZyme^®^ G5000, FibreZyme^®^ LBL CONC, FibreZyme^™^ LDI and FibreZyme^™^ G4 (Dyadic)	Modify cellulose and hemicellulose components of virgin and recycled pulps
	Amylase	Dexamyl-HTP (Varuna Biocell)	Modification of starch of coated paper

#### Hot Extremozymes Applied in the Food and Beverage Market

Since 1973, the starch-processing industry has grown into one of the largest markets for enzymes. Enzymatic starch hydrolysis is used to form syrups through liquefaction, saccharification, and isomerization processes. Products of starch hydrolysis, such as glucose, maltose, and oligosaccharides, can be used for the production of a variety of non-food products including alcohols, polyols, ascorbic acid, lysine, etc. In the manufacture of syrups, liquefaction and saccharification processes are run at high temperatures, easily over 60–70°C (Bentley and Williams, 1996). α-amylases are used during the liquefaction step to form maltodextrins and mash viscosity prior to saccharification and fermentation with yeast. Novozymes Avantec^®^, Termamyl^®^ SC and Liquozyme^®^ products liquefy and prepare the starch source for optimal fermentation, resulting in higher yields and reduced operating costs. During the saccharification step, pullunases and glucoamylases like Spirizyme^®^ from Novozymes, are used on liquefied starch-containing substrates to produce sugars for the fermentation at 70°C. These hot extremozymes allow for maximum starch conversion and consistent fermentation across variable mash and plant conditions. Pullunases and glucoamylases are used for producing glucose syrups, and pullunase and β-amylase for producing maltose syrups. The thermophilic α-amylase Fuelzyme^®^, developed and marketed by Verenium Corporation (San Diego, CA, USA), a division of BASF (Mannheim, Germany), is used for obtaining superior reduction in mash viscosity when starch liquefaction is performed at low pH. Fuelzyme^®^ performs at a temperature of 88–91°C and is also used to increase fuel ethanol yields as a result of improved starch conversion.

Commonly, enzymes isolated from hyperthermophiles work optimally between 80 and 110°C and at pH levels from 4.0 to 7.5. These conditions correlate with the optimal conditions for starch liquefaction (100°C and pH 4.0–5.0) (Niehaus et al., 1999). Thus, characterization and development of novel hyperthermophilic enzymes is essential for these industrial processes. The expression of a mutant thermostable α-amylase from *B. licheniformis*, with optimum activity at high temperature and lower pH, in *E. coli* and *P. pastoris*, was recently achieved (Du et al., 2006). Because of their high-temperature activity profiles, β-amylases from *Thermoanaerobacterium thermosulfurigenes* and *Clostridium thermocellum* SS8 are also good candidates for saccharification processes (Kitamoto et al., 1988; Swamy et al., 1994). However, further research into new β-amylases is still needed.

In recent years, amylopullulanases have emerged as useful starch-processing enzymes for the production of maltose and maltotriose syrups. Amylopullulanases are sparking a new trend of replacing α-amylases and β-amylases in the conventional starch liquefaction process, because of their bi-functionality for catalyzing debranching and liquefying reactions, and because of their calcium independence. In addition to their applications in industrial starch conversion, the previously mentioned amylopullulanases features make them useful as detergent additives. Several thermophilic amylopullulanases have been discovered and cloned into microorganisms like *E. coli* and *B. subtilis* for increased expression (Nisha and Satyanarayana, 2013). A starch saccharification process using only amylolytic enzymes (α-amylase, amylopullulanase, and glucoamylase) from thermophiles was described in 2004 (Satyanarayana et al., 2004).

Glucose (xylose) isomerases catalyze the isomerization of glucose into fructose for the preparation of fructose-based syrups. The conversion rate of glucose-fructose is altered at high temperatures between 60 and 90°C. Sweetzyme^®^ from Novozymes is an immobilized glucose isomerase from *Streptomyces murinus* that is able to perform at temperatures from 55 to 60°C. Other xylose isomerases have been characterized from *Thermotoga neapolitana*, *Thermotoga maritima*, *Thermus aquaticus*, and *Thermus thermophilus* (Vieille et al., 1995) among other microorganisms. These are not yet commercially available. Recent reports have described thermophilic glucose isomerases isolated from *Fervidobacterium gondwanense* and *Bacillus sp*. isolated from agricultural soil, with optimal temperatures for activity of 70°C, making both enzymes good candidates for industrial applications (Kluskens et al., 2010; Sukumar et al., 2013).

Amylases are also applied in processes, such as baking, brewing, the preparation of digestive aids, and in the production of cakes and fruit juices. Currently, Novamyl^®^ (Novozymes) a thermostable maltogenic amylase of *Bacillus stearothermophilus* is used commercially in the bakery industry for improved freshness and other bread qualities. AlphaStar PLUS is a food grade, bacterial α-amylase from *B. subtilis* used in brewing applications. These enzymes are endoamylases, capable of randomly hydrolyzing the predominating *a*-d-(1 → 4) glucosidic linkages of starch to rapidly reduce the viscosity of gelatinous starch. It is stable up to 90°C. GlucoStar PLUS (Dyadic International) is an industrial amyloglucosidase liquid product used in processing aids which is derived from a non-genetically modified strain of *A. niger*. The enzyme α-amylase is used to break down both components of starch, amylose and amylopectin, as well as the resultant products of their dextrinization. CeluStar XL (Dyadic International) is an enriched preparation that contains xylanase, cellulase, β-glucanase, pectinase, mannanase, xyloglucanase, laminarase, β-glucosidase, β-xylosidase, α-l-arabinofuranosidase, amylase, and protease to hydrolyze hemicellulose and cellulose to lower molecular weight polymers under the proper processing conditions. This product is used in brewing, typically within a broad range of pH (3.5–7.5) and temperature 35 and 60°C. Other thermophilic enzymes developed by Dyadic International, mainly applied in the brewing industry, are BrewZyme LP, Dyadic Beta Glucanase BP CONC, Dyadic xylanase PLUS, Xylanase 2XP CONC, AlphaStar CONC, Protease AP CONC, and Protease PLUS.

Panzea BG and Panzea 10X BG of Novozymes, are both pure xylanases from *B. licheniformis* used in the bakery industry. These products aid in the large-scale manufacture of dough with the desired texture and appearance. Other hot extremozymes from Novozymes with applications in the food industry are Fungamyl^®^ (α-amylase), which is used to improve the color and volume of bread, Lipopan^®^ (lipase) and Pentopan^®^ (xylanase) which are used to obtain stronger dough and Gluzym^®^ (glucose oxidase) which is used to obtain stronger gluten.

#### Hot Extremozymes Applied in Pulp and Paper

Pulp is a cellulosic fibrous material made from wood, fiber crops, and waste paper. Commonly, pulp is made by chemical and mechanical procedures that separate cellulose fibers from the rest of the components of wood (hemicellulose and lignin). These methods include applying harsh conditions like extremely high temperatures (in many cases over 80°C), alkaline pHs and the use of strong chemicals (sodium sulfide, sodium hydroxide, and chlorine). In efforts to complement the current pulping procedures, enzymatic bio-pulping is becoming an attention-grabbing approach as it offers an eco-friendly, safer, and profitable solution for the pulp and paper industry. The utilization of stable hyperthermophilic/alkaline enzymes represents a valuable addition to the current pulping processes by increasing the efficiency and reducing the use of dangerous chemicals. Despite the fact that currently the market for enzymes in the pulping and paper industry is small, it is expected to reach a size of $261.7 million in 2018, with a CAGR of 7% (2013–2018). Europe is positioned as the main geographical market (Dewan, 2014).

Among the enzymes related to bio-pulping, xylanases are considered of the highest impact. They can break down the hemicellulose from plant cell walls by degrading the linear polysaccharide β-1,4-xylan into xylose. In this manner, xylanases facilitate the release of lignin, in a process called bio-bleaching. Hyperthermophilic xylanases have been isolated from a number of different microorganisms, such as *T. maritima*, *Streptomyces sp*., *Thermotoga thermarum*, and *Thermoascus aurantiacus*. Some of these enzymes have already been tested in bio-bleaching processes (Chen et al., 1997; Beg et al., 2000; Milagres et al., 2004; Jiang et al., 2006; Shi et al., 2013). However, few xylanases are currently available in the market for pulp and paper applications. Luminase^®^ PB-100 and PB-200, for example, are highly thermostable xylanases supplied by Verenium Company. Luminase^®^ PB-100 acts between 40 and 70°C, while Luminase^®^ PB-200 can be used between 60 and 90°C. Megazyme (Bray, Ireland) offers Xyn 10A, a recombinant endo-1,4-β-d-xylanase from *T. maritime*, which has an optimal temperature of 80°C but displays temperature stability up to 90°C, and a pH stability up to pH 9.

Laccases are also applied in the bio-bleaching of pulp. By directly degrading lignin, the substance that gives a dark color to pulp, laccases increase the brightness of the final product. In addition, laccases have been observed to play a role in the removal of lipophilic extractives which are part of the problem of pitch deposition (Virk et al., 2012). Several laccases have been described for use in bio-bleaching, particularly from fungal origin since bacterial laccases are not well characterized (Baldrian, 2004; Virk et al., 2012). Novozymes commercialize a recombinant thermostable laccase from the fungus *Myceliophthora thermophila* which has been cloned in *A. oryzae* and is active up to 70°C (Xu et al., 1996; Berka et al., 1997) In 2006, AB Vista (Wiltshire, UK) was granted a patent for a thermostable laccase enzyme which is effective at temperatures between 30 and 80°C (Paloheimo et al., 2006). Even though there are certain commercial options, it is necessary to discover and develop novel laccases that can perform optimally under the harsh conditions of the pulping process. A recent report described the bio-bleaching of wheat straw pulp using a recombinant laccase from the hyperthermophilic bacterium *Thermus thermophiles*, which performs optimally at 85°C (Zheng et al., 2012).

Hyperthermophilic lipases are applied for pitch control in the paper production process. Pitch generates sticky deposits which affect mill operations and the quality of the final product. Lipases degrade the triglycerides of pitch reducing its deposition. Novozymes offers Resinase^®^ HT, a lipase with temperature stability up to 90°C and pH stability between pH 5 to 9. This enzyme was developed by directed evolution of an older Resinase^®^ variant and is one of the most used enzymes for pitch control in the pulp and paper industry in North America, China, Japan, and other countries (Gutierrez et al., 2009). In addition to lipases, novel hyperthermophilic esterases have been suggested to improve pitch control, by degrading other sticky compounds, such as adhesive and coatings which contain significant amounts of esters (Calero-Rueda et al., 2004). Optimyze^®^, developed by Buckman Laboratories (Memphis, TN, USA) is an enzyme-mix which contains an esterase that breaks up ester bonds of polyvinyl acetate with activity up to a temperature of 60°C. Novel hyperthermophilic lipases and esterases with higher optimal temperatures and more alkaline pHs are needed to solve the pitch problems of the pulp and paper industry.

Other hyperthermophilic enzymes that find application in the pulp and paper industry are cellulases. These enzymes increase the brightness and strength properties of paper sheets and the overall efficiency of the refining process (Kuhad et al., 2011). In 2012, Dyadic International launched FibreZyme^®^ G500, a cellulase with a wide temperature and pH range for activity, from 35 to 75°C and pH 4.5 to 9. This is an adaptable product for most pulping procedures. In addition, a recent report described a novel recombinant archaeal cellulase with an optimal activity at 109°C, a half-life of 5 h at 100°C, and it is highly resistant to strong detergents, high-salt concentrations, and ionic liquids (Graham et al., 2011).

Even though many advances have been made toward developing novel enzymes for the pulp and paper industry, additional efforts are needed to discover and develop high-performance enzymes that are better adapted to the current conditions of the pulping industry. In addition to xylanases, lipases, esterases and cellulases, hyperthermophilic pectinases (improve retention in mechanical pulps), and amylases (finishing and coating of paper) will further aid in the implementation of environmentally friendly and more efficient procedures for the pulp and paper industry (Reid and Ricard, 2000; de Souza and de Oliveira Magalhaes, 2010).

## Challenges and Conclusion

Despite the inherent advantages of extremozymes, the actual number of available extremophilic bio-catalytic tools is very limited. Working with extremophiles and/or extremozymes requires the adaptation and creation of new methodologies, assays, and techniques that operate under non-standard conditions. Many of the tools that are currently used in classical microbiology and biochemistry experimentation cannot be applied toward extremophilic research because they do not possess the chemical and/or mechanical properties to withstand extreme conditions. Similarly, techniques for researching common microorganisms need to be further adjusted to fit the requirements of extremophiles. A classic example is the plating of hyperthermophiles on a solid surface. Conventional streaking on agar-based media is impracticable because agar melts and water evaporates quickly at such high temperatures. Alternative solidifying agents are used to grow thermophiles and hyperthermophiles, such as silica gel, starch, and Gelrite, a low-acetyl gellan gum made from *Pseudomonas*. Additionally, the large technical gap between producing an enzyme under laboratory conditions and obtaining a final commercializable product is still a problem for the development of novel biocatalysts. Several scientific challenges need to be solved before it will be possible to fully realize the potential of extremozymes.

Nature provides a vast source of biocatalysts. However, the probability of finding the right enzymatic activity for a particular application depends on the available technical capabilities to efficiently assess this large biodiversity. This capability is mainly mediated by technologies, such as metagenomic screenings, genome mining, and direct enzymatic exploration (Leis et al., 2013; Adrio and Demain, 2014; Bachmann et al., 2014).

Metagenomic screenings and genome mining are commonly based on the presence of DNA/RNA sequences coding for determined enzymes. These methods require that the search for a novel enzyme is based on genetic sequence homology to already described enzymes. This creates a bias and hinders the discovery of truly new enzymes. The discovery of new enzymes based on genetic sequences also does not always give accurate information, especially for less studied organisms like extremophiles. To get industry-quality results for new enzymes, these approaches require further processing through directed evolution and protein engineering. Also, in the case of functional metagenomic screenings, there are several technical challenges that need to be addressed. For instances, the isolation of high-quality DNA from environmental samples, which often is contaminated with humic acids, the difficulties to lyse extremophilic microorganisms, the cell recovery biases, and the need of appropriate hosts for heterologous expression of recovered genes from metagenomics data.

An alternative method is presented in the direct exploration of extremophilic enzymes based on functional screenings of enzymatic activities in large collections of microorganisms. The functional detection of an enzymatic activity determines the existence of the target biocatalyst in the sample, and its behavior under previously determined conditions. It is important to note that confirming the presence and the functionality of a biocatalyst, under the actual conditions of a particular industrial process, is an important step toward further developing an industrial product. There are also drawbacks to this approach. To test the presence of an enzyme in the crude extract of a microorganism, extremely robust enzymatic assays needs to be developed and implemented. These then have to be adapted to the required conditions for enzyme activity. Also, in order to make functional detection more appealing for industry purposes, it is absolutely required that this approach is further developed to handle hundreds of samples simultaneously under small-scale and automated conditions to allow for high-throughput discovery of efficient biocatalysts for a specific application. Developing automated systems for working with extremophiles and extremozymes is not only a scientific challenge, but a technological and engineering one as well. The task is not only related to media composition or assay development, it also involves developing tools and instruments that can withstand and function optimally under extreme conditions. Currently, High-Throughput Screening (HTS) technologies are being implemented by pharmaceutical companies for identifying new drugs and chemicals (Zhu et al., 2010). The application of these technologies toward the search for novel biocatalysts may be the solution to some of these problems (Donadio et al., 2009; King et al., 2010; Glaser and Venus, 2014). Additionally, the application of novel culture techniques for uncultured bacteria, such as the recently reported iChip (Ling et al., 2015) will further aid in the identification and development of novel industrial enzymes.

Technical limitations are not only found at the moment of discovering novel biocatalysts, but also when the biocatalysts are fine-tuned for industrial applications. Using extremophiles directly as the producing microorganism for extremozymes is an ideal situation, but it presents several difficulties. Operating bioreactors under extreme conditions, such as high and low pHs, high temperatures or high concentration of salts, shortens the lifetime of sensors and seals in the bioreactors. Also, in many cases, extremophiles do not grow optimally in bioreactors. In the case of (hyper)thermophiles this problem has been attributed to the accumulation of toxic compounds as a result of Maillard reactions (a series of complex reactions between reducing sugars and amino acids, occurring at high temperatures), as described in the cultivation of *Aeropyrum pernix* (Kim and Lee, 2003). One of the alternatives to overcome the low biomass yields is to appropriately design the culture medium. Although there are examples where a defined synthetic medium is used for the cultivation of extremophiles (Biller et al., 2002), complex culture medium containing yeast extract and peptone is, by far, the most commonly employed for cultivation of extremophiles (Dominguez et al., 2004; Fucinos et al., 2005a,b).

Due to the limitations of growing extremophiles for producing extremozymes, the current strategy used is to clone and express the genes encoding the desired product in mesophilic hosts prior to the operation in a bioreactor (Karlsson et al., 1999). Many thermophilic genes have been cloned and expressed in mesophilic hosts, yielding highly active and temperature stable enzymes, such as the thermoalkalophilic lipase from *Bacillus thermocatenulatus* (Rua et al., 1998). However, recombinant expression of (hyper)thermophilic enzymes in *E. coli* and other bacterial hosts is still problematic. Expressing genes from archaea, for example, in these mesophilic host organisms can lead to misreading genes. This is not the case with bacterial genes, making them better candidates for cloning into bacterial hosts (Kristjansson, 1989). There is a clear need for new hosts able to properly express hyperthermophilic archaeal enzymes, such as the recent efforts reported for the expression of hyperthermophilic cellulases of the archaea *Pyrococcus sp* in the fungus *Talaromyces cellulolyticus* (Kishishita et al., 2015). The development of novel culture and molecular tools, more efficient mass production processes, and novel technologies for genetic and protein engineering will further advance the application of extremozymes in different industries.

## Conflict of Interest Statement

The authors declare that the research was conducted in the absence of any commercial or financial relationships that could be construed as a potential conflict of interest.

## Footnotes

^1^http://www.marketsandmarkets.com/PressReleases/specialty-enzymes.asp

^2^http://www.unilever.com/sustainable-living-2014/reducing-environmental-impact/greenhouse-gases/reducing-ghg-in-consumer-use/index.aspx

^3^http://www.pg.com/en_US/downloads/sustainability/reports/PG_2014_Sustainability_Report.pdf

^4^http://www.swissaustral.com
